# Temporal stability and geographical divergence of skin microbiota in a treefrog: insights from microbial communities and volatile secretions

**DOI:** 10.1093/ismeco/ycag125

**Published:** 2026-05-07

**Authors:** Tao-Yue Chen, Ke Deng, Qiao-Ling He, Tong-Liang Wang, Ji-Chao Wang, Jian-Guo Cui

**Affiliations:** Mountain Ecological Restoration and Biodiversity Conservation Key Laboratory of Sichuan Province, Chengdu Institute of Biology, Chinese Academy of Sciences, Chengdu, Sichuan Province 610213, China; University of Chinese Academy of Science, Beijing 101408, China; Mountain Ecological Restoration and Biodiversity Conservation Key Laboratory of Sichuan Province, Chengdu Institute of Biology, Chinese Academy of Sciences, Chengdu, Sichuan Province 610213, China; Mountain Ecological Restoration and Biodiversity Conservation Key Laboratory of Sichuan Province, Chengdu Institute of Biology, Chinese Academy of Sciences, Chengdu, Sichuan Province 610213, China; University of Chinese Academy of Science, Beijing 101408, China; Ministry of Education Key Laboratory for Ecology of Tropical Islands, Key Laboratory of Tropical Animal and Plant Ecology of Hainan Province, College of Life Sciences, Hainan Normal University, Haikou, Hainan Province 571158, China; Ministry of Education Key Laboratory for Ecology of Tropical Islands, Key Laboratory of Tropical Animal and Plant Ecology of Hainan Province, College of Life Sciences, Hainan Normal University, Haikou, Hainan Province 571158, China; Mountain Ecological Restoration and Biodiversity Conservation Key Laboratory of Sichuan Province, Chengdu Institute of Biology, Chinese Academy of Sciences, Chengdu, Sichuan Province 610213, China; University of Chinese Academy of Science, Beijing 101408, China

**Keywords:** geographical variation, *Kurixalus hainanus*, skin microbiota, temporal variation, volatile secretions

## Abstract

Skin microbiota, which is highly sensitive to environmental heterogeneity, together with skin secretions, plays a critical role in defense and communication in amphibians. However, the temporal and geographical patterns of skin microbiota, as well as the composition of the skin volatile secretions, remain largely unexplored in many amphibian species. In the present study, we collected skin bacterial samples from the Hainan frilled tree frog (*Kurixalus hainanus*) in the Lingshui population across four consecutive months (April–July) to assess temporal dynamics, and additionally sampled frogs from the Jinxiu and Shanglin populations to examine geographical differentiation. Skin volatile secretions were also characterized and compared among the three populations. The results showed that temporal variation in skin microbiota was primarily driven by a small fraction of rare taxa, whereas the overall evenness and dominant components of the core skin microbiota remained relatively stable over time. In contrast, pronounced differentiation in microbiota composition was observed among populations, indicating a strong influence of habitat heterogeneity. We also found that skin secretions were largely similar among the three populations. Decanal and 1-octanol were identified as candidate compounds underlying disturbance odors in *K. hainanus*, and three terpenoid compounds detected exclusively in individuals from the Shanglin population may be derived from environmental sources or synthesized by the resident skin microbiota. Together, our findings characterize the temporal and spatial variation of skin microbiota in *K. hainanus*, while simultaneously identifying population-level variation in skin volatile secretions among populations and establishing a framework for future research to address the interplay between these two critical skin components.

## Introduction

Animals harbour diverse, long-term symbiotic microbial communities (both internally and externally), which play essential roles in host survival. Among these, the gut microbiota has been intensively studied for its roles in nutrient absorption [1, 2], intestinal barrier formation [3, 4], and ecological adaptation [5], and the modulation of host neural and behavioral functions through the gut–brain axis [6]. Despite the critical importance of the gut microbiota, its composition and diversity are not static but are influenced by multiple factors, including host genetics, diet, microbial interactions, and environmental variables [3, 7–9]. Environmental influences are generally indirect and host-mediated, as the intestinal mucosal barrier largely limits direct exposure of gut microbes to external environmental variation.

In contrast, the skin microbiota is in direct contact with the surroundings and thus highly sensitive to environmental variation [10, 11], such as temperature [12], ultraviolet radiation [13], water quality [14], and social interactions [15]. For example, elevated water temperatures increase stress markers in the skin mucus of cold-water sturgeon, leading to a decline in beneficial bacteria and an increase in pathogenic ones [12]. The skin microbiota is not only environmentally sensitive but also functionally important. Many studies have shown that a key function of skin microbiota is immune defense, and that environmental changes can alter community composition, potentially leading to a sharp increase in host susceptibility to infection [16]. In addition, the skin microbes can participate in the production and transformation of chemical compounds that mediate species recognition, sex recognition, and even individual recognition [17–19]. For example, Theis et al. [20] reported that skin microbes produce species-specific smells in spotted hyenas (*Crocuta crocuta*) and striped hyenas (*Hyaena hyaena*) and sex-specific smells in both species. A recent study suggested that alterations at the skin surface, including changes in local immunity, secretions, and associated microbes, can influence host behavior via neuroimmune and chemosensory pathways, in a manner comparable to the gut–brain axis [21]. Therefore, environmentally driven variation in skin microbiota and its associated chemical secretions may play an important role in host health and ecological adaptation.

The skin microbiota and chemical secretions constitute two fundamental systems for host defense and communication that facilitate adaptation to environmental conditions. This is particularly prominent in amphibians. Amphibians rely on a thin, moist, and mucous-covered skin to maintain physiological homeostasis [22] and to provide a sugar-rich substrate supporting abundant microbial growth. These microbial communities play essential roles in resisting pathogens (especially *Batrachochytrium dendrobatidis*) [23, 24], promoting wound healing [25], and adapting to polluted environments [26]. Accordingly, the skin microbiota serves as a primary line of defense. Furthermore, microbial metabolic activities may contribute to the synthesis and transformation of skin secretions [27]. In turn, these volatile secretions play important roles in intraspecific communication, such as mate recognition and alarm signaling [28], as well as in interspecific defense, including predator deterrence [29] and pathogen inhibition [30]. Together, the microbiota and secretions form an integrated surface shield that enables amphibians to cope with complex environmental conditions. However, the temporal and geographical variation in skin microbiota, as well as the composition of skin volatile secretions, remain largely unexplored in many amphibian species.

The Hainan frilled tree frogs (*K. hainanus*) belong to the family Rhacophoridae and the genus *Kurixalus* [31], and this species is distributed in Guangxi, Guangdong, Guizhou, and Hainan Provinces in China [32]. At Diaoluo Mountain National Nature Reserve in Lingshui County, Hainan Province, China (Latitude: 18.72°N, Longitude: 109.87°E, Elevation: 933 m), *K. hainanus* is a dominant species and breeds primarily between April and August [33–35]. In this study, we collected skin bacteria from the Lingshui population between April and July to examine whether the composition and diversity of skin microbiota exhibit temporal variation. We also collected samples from populations in Jinxiu County and Shanglin County, Guangxi Province, to determine whether skin microbiota exhibit geographical variation. Since skin bacteria are influenced by environmental conditions [36], we hypothesized that *K. hainanus* would show both temporal and spatial variation in skin microbiota. In addition, previous studies have shown that males adjust their call rate or call frequency, and females modify their mate choice decision in response to conspecific volatile secretions [37–39]. To further investigate the composition and potential functions of skin volatile secretions, we identified and compared skin secretions of *K. hainanus* among the three populations.

## Materials and methods

### Skin microbiota sampling

Skin microbiota of *K. hainanus* was collected from three populations across different localities. These three localities represent distinct climatic conditions in southern China, ranging from a tropical monsoon climate in the montane rainforests of Lingshui (Hainan Island) to subtropical monsoon climates in mountainous (Jinxiu) and lower-elevation karst (Shanglin) regions of Guangxi Province. To investigate temporal variation in the composition and diversity of skin microbiota, we collected seven individuals per month from April to July 2021 from the Lingshui population in Hainan Province (18.72°N, 109.87°E; *n* = 28), with all sampling conducted directly in the field. For the spatial comparison, samples from the Jinxiu (24.12°N, 110.21°E; *n* = 7) and Shanglin (23.24°N, 108.63°E; *n* = 7) populations in Guangxi Province were collected in July 2022. Frogs and associated vegetation from these two populations were transported to the Chengdu Institute of Biology, Sichuan Province, where all sampling procedures were completed on the same day. To ensure comparability, only the seven samples collected in July from the Lingshui population were included in the analysis of geographical variation.

Sampling was conducted between 20:00 and 22:00, and all focal individuals were collected while actively calling at breeding sites. Accordingly, all sampled frogs were adult males in breeding condition, and only healthy individuals without visible skin lesions were included. Before sampling, each frog was rinsed three times with sterile water to remove potential transient bacteria [11]. Skin microbiota was then collected using sterile, non-germicidal swabs by wiping the dorsal, ventral, and lateral skin of the frogs, with a minimum of 30 swabbing strokes per individual [40]. The swabs were placed into sterile EP tubes and immediately frozen in liquid nitrogen or stored at −80°C until DNA extraction. All procedures were performed using sterile gloves to avoid contamination.

### 16S rRNA gene sequencing and statistical analyses of microbiota

Total genomic DNA was extracted from the frog’s skin samples using the TGuide S96 Magnetic Soil /Stool DNA Kit (Tiangen Biotech, Beijing, Co., Ltd.) according to the manufacturer’s instructions. The quality and quantity of the extracted DNA were examined using electrophoresis on a 1.8% agarose gel, and DNA concentration and purity were determined with NanoDrop 2000 UV–Vis spectrophotometer (Thermo Scientific, Wilmington, USA). The hypervariable region V3-V4 of the bacterial 16S rRNA gene was amplified with primer pairs 338F: 5′- ACTCCTACGGGAGGCAGCA-3′ and 806R: 5′- GGACTACHVGGGTWTCTAAT-3′. Both the forward and reverse 16S primers were tailed with sample-specific Illumina index sequences to allow for deep sequencing. The PCR was performed in a total reaction volume of 10 μl: DNA template 5–50 ng, forward primer (10 μM) 0.3 μl, reverse primer (10 μM) 0.3 μl, KOD FX Neo Buffer 5 μl, dNTP (2 mM each) 2 μl, KOD FX Neo 0.2 μl, and finally ddH2O up to 10 μl. After initial denaturation at 95°C for 5 min, followed by 20 cycles of denaturation at 95°C for 30 s, annealing at 50°C for 30 s, and extension at 72°C for 40 s, and a final step at 72°C for 7 min. The amplified products were purified with the Omega DNA purification kit (Omega Inc., Norcross, GA, USA) and quantified using Qsep-400 (BiOptic, Inc., New Taipei City, Taiwan, ROC). Prior to sequencing, each library was adjusted according to its concentration, and an equal amount of DNA from each sample library was pooled to construct the final sequencing library. The amplicon library was paired-end sequenced (2 × 250) on an Illumina Novaseq 6000 (Beijing Biomarker Technologies Co., Ltd., Beijing, China). According to the quality of single nucleotide, raw data were primarily filtered by Trimmomatic (version 0.33) [41]. Identification and removal of primer sequences was processed by Cutadapt (version 1.9.1) [42]. PE reads obtained from previous steps were assembled by USEARCH (version 10) [43] and followed by chimera removal using UCHIME (version 8.1) [44]. The raw read counts for each sample are listed in Table S1. The high-quality reads generated from the above steps were used in the following analysis. Sequences with similarity >97% were clustered into the same operational taxonomic unit (OTU) by USEARCH (version10) [43], and the OTU counts less than 2 in all samples were filtered. Taxonomy annotation of the OTUs was performed based on the Naive Bayes classifier in QIIME2 using the SILVA database (release 138.1) [45] with a confidence threshold of 70%. The raw sequences were deposited in the NCBI Sequence Read Archive (SRA) database under BioProject accession number PRJNA1399319.

The temporal and spatial analyses were conducted on different subsets of samples, so the OTU tables were rarefied separately for each dataset to 30 000 reads per sample before calculating alpha and beta diversity. Alpha diversity, including ACE, Chao 1, Shannon, and Simpson indexes, was calculated using QIIME2 software. We first tested for overall differences using the Kruskal–Wallis test, followed by pairwise Wilcoxon rank-sum tests with Benjamini–Hochberg false discovery rate (FDR) adjustment, with *P* < .05 indicating a significant difference. Beta diversity was estimated using Bray–Curtis dissimilarity metric and visualized by principal coordinate analysis (PCoA) to assess differences in community composition among samples. Permutational multivariate analysis of variance (PERMANOVA) was used to test for significant differences in beta diversity between samples from different groups. Differences in genus-level relative abundances across months were assessed using the Kruskal–Wallis test, followed by Nemenyi post-hoc pairwise comparisons when significant differences were detected (corrected *P* < .05). Linear discriminant analysis (LDA) coupled with effect size (LEfSe) was applied to evaluate the differentially abundant taxa. We calculated prevalence and mean relative abundance for each genus. Prevalence is defined as the proportion of samples in which the genus appears with a relative abundance greater than 0. Core taxa were defined as those with a prevalence of ≥80% and a mean abundance of ≥0.1%. Conversely, rare taxa were classified as those with a prevalence of <20% and a mean abundance of <0.1% [46]. We also examined the temporal variation in relative abundance of core community and rare community with the Kruskal-Wallis test, respectively.

### Volatile secretions sampling

Volatile secretions sampling and analysis were conducted from June to August 2022. A total of 11, 10, and 7 male frogs were captured from the Lingshui, Jinxiu, and Shanglin populations, respectively. All individuals and vegetation from their microhabitats were carefully transported to the Chengdu Institute of Biology, Sichuan Province, for volatile secretions sampling.

Volatile secretions were sampled from skin using headspace solid-phase microextraction (HS-SPME) according to the following procedure [29]. The frogs were rinsed three times with sterile water and euthanized by immersion in liquid nitrogen. Then, two or three specimens were transferred to a dissecting tray for thawing to room temperature. The whole skin of these specimens was rapidly excised using stainless steel scissors and tweezers, and immediately placed in a ceramic mortar containing liquid nitrogen to minimize the loss of components [47]. The skins were homogenized and transferred into a headspace vial of 20 ml, which was sealed with aluminum crimp caps. Subsequently, an extraction fiber (50/30 μm, DVB/CAR/PDMS, SUPELCO, Bellefonte, PA, USA) was inserted into the vial, and the samples were maintained in a water bath at 55°C for a 40-minute collection period. Upon completion of the collection, the extraction fiber was promptly removed for subsequent experimental procedures.

### Chromatographic analysis

After sampling, the extraction fiber was immediately inserted into the injection port of a gas chromatography coupled to a Mass Spectrometer Detector (GC/MS A91Plus-AMD10, Panna Instruments, Changzhou, Co., Ltd). The column oven temperature program was set as follows: initial temperature of 40°C held for 3 minutes; subsequently ramped up to 230°C at a rate of 7°C/min; finally increased to the final temperature of 270°C at 50°C/min and maintained for 3 minutes. High-purity helium (99.999%) was used as the carrier gas with a constant flow rate of 1 ml/min. The injector operated in splitless mode at a constant temperature of 250°C. The mass spectrometer was set to scan ions within the range of 35–550 amu. Upon inserting the extraction fiber into the injection port, the extraction phase was first pushed out for desorption (5 minutes) to release the adsorbed chemical substances into the instrument. Subsequent aging (10 minutes) was performed to eliminate residual chemicals on the fiber. Finally, the extraction fiber was removed, and the instrument was allowed to proceed with the analysis run. All samples, including the blank controls, were run under the same chromatographic conditions.

Data analysis was performed using Q-Tek Maestro Software Kit, which includes the NIST17 standard mass spectral library. Compounds were tentatively identified when their mass spectral similarity to the reference library entries was ≥80%. After excluding chemical substances frequently detected in the blank control group, volatile compounds previously reported in frogs based on literature were selected as target analytes [29, 47]. All samples were monitored using the selected ion monitoring method described in Smith et al. [48]—specifically, three major characteristic ion peaks of each target analyte were selected to locate the corresponding compounds in the chromatograms—for further data extraction, analysis, and confirmation of the target compounds. All chemical substances were named according to the IUPAC nomenclature and classified using Classyfire, a program that automatically matches known compounds based on chemical structures and structural features, incorporating a taxonomy consisting of no fewer than 4800 distinct categories [49].

## Results

### The temporal variation of skin microbiota in *K. hainanus*

The average number of OTUs per sample was 2519 (range: 2223–3231) in 28 skin samples of *K. hainanus* collected from the Lingshui population. These OTUs were classified into 46 phyla, 119 classes, 342 orders, 764 families, and 1996 genera. Rarefaction curves approached saturation, indicating that sequencing depth was sufficient for the analyses performed (Fig. S1).

**Figure 1:**
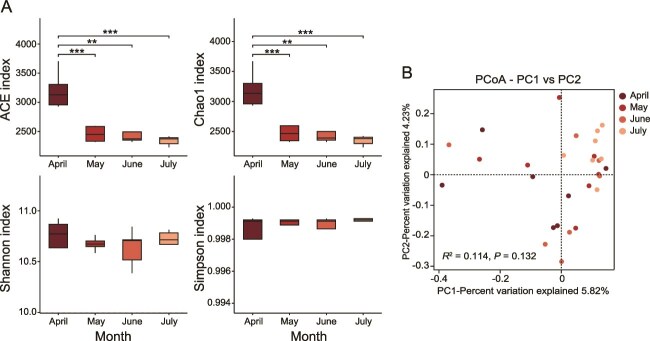
Skin microbiota diversity of *Kurixalus hainanus* from April to July. (A) Alpha diversity based on ACE, Chao 1, Shannon, and Simpson indices. Differences were tested by Kruskal–Wallis test followed by pairwise Wilcoxon rank-sum tests with Benjamini–Hochberg FDR correction. (B) PCoA based on bray-Curtis distances. PERMANOVA was used to assess differences among months. Asterisks indicate significant differences between months. ^*^P < .05, ^**^P < .01, ^***^P < .001.

For alpha diversity of the skin microbiota, the ACE and Chao1 indices exhibited significant differences among four months (Kruskal–Wallis test: *P* < .05, Fig. 1A). Specifically, the ACE and Chao1 indices in April were significantly higher than those in May, June, and July (Wilcoxon rank-sum tests: all *P* < .05, Fig. 1A). In contrast, there was no significant difference in Shannon or Simpson indexes of the skin microbiota among 4 months (Kruskal–Wallis test: *P* > .05, Fig. 1A). For beta diversity of the skin microbiota, PCoA analysis based on community composition did not reveal distinct clustering of samples among the four months. The first two principal coordinates (PC1 and PC2) explained 5.82% and 4.23% of the total variance, respectively (Fig. 1C). Accordingly, the PERMANOVA analysis showed that the effect of month on community composition was not significant (Bray-curtis: *R*^2^ = 0.114, *P* = .132, Fig. 1B).

**Figure 2:**
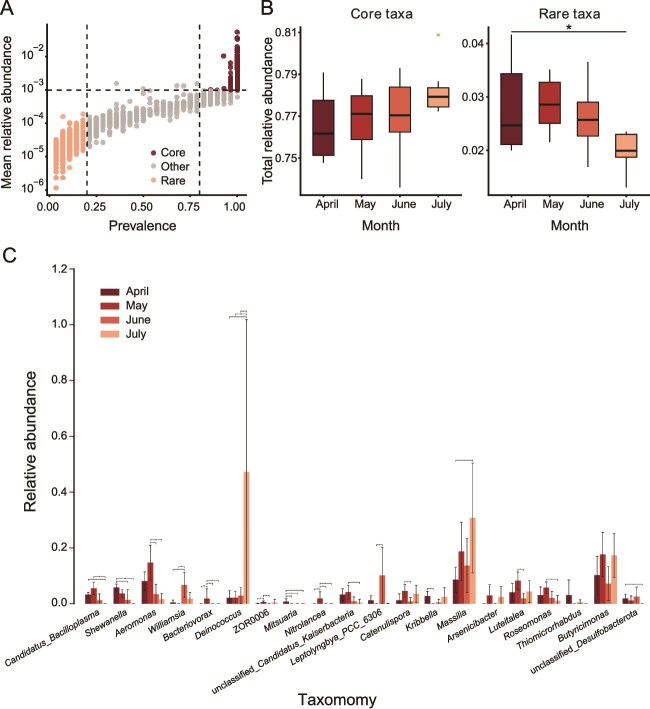
Temporal variation in skin microbiota of *Kurixalus hainanus* from April to July at genus level. (A) Prevalence-abundance scatter plot showing the separation between core taxa (prevalence ≥80%, mean relative abundance ≥0.1%) and rare taxa (prevalence <20%, mean relative abundance <0.1%). (B) Relative abundance of core and rare taxa. (C) The top 20 genera with significant changes in relative abundance. Differences were analyzed using the Kruskal–Wallis test followed by Nemenyi post hoc pairwise comparisons when significant differences were detected (corrected *P* < .05). Asterisks indicate significant differences between months. ^*^P < .05, ^**^P < .01, ^***^P < .001.

The prevalence-abundance scatter plot visually demonstrated the distinct separation between core and rare taxa, validating the predefined classification standards (Fig. 2A). Core taxa exhibited relatively stable total abundance across the four months, with no significant differences detected (Kruskal-Wallis test: *P* = .32, Fig. 2B), whereas rare taxa displayed significant temporal variation in total abundance (Kruskal-Wallis test: *P* = .0486, Fig. 2B). The Kruskal–Wallis test identified genera exhibiting significant differences in abundance across months (corrected *P* < .05). The top 20 genera with the smallest p-values were shown in Fig. 2C, and each exhibited a relative abundance of less than 1.2%. The relative abundance of *Deinococcus* in July was significantly higher than that in April, May, and June (all *P* < .05, Fig. 2C). The relative abundance of *Aeromonas* increased in May and decreased gradually from June to July (Fig. 2C). Although the overall abundance of *Mitsuaria* was low, its relative abundance in April was significantly higher than that in the other months (all *P* < .05, Fig. 2C). The LEfSe analysis (|LDA| > 3) further supported these temporal differences, with Deinococcota-related taxa being consistently enriched in July, including *Deinococcus*, Deinococcaceae, Deinococci, and Deinococcales, suggesting that this lineage may serve as a robust marker of July-associated microbiota.

**Figure 3:**
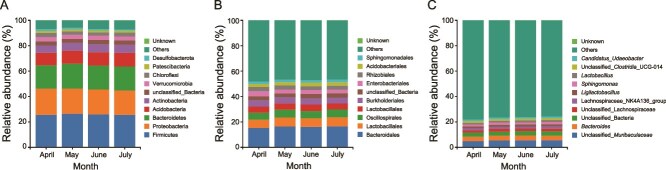
Relative abundance of skin microbiota of *Kurixalus hainanus* from April to July at phylum (A), orders (B), and genus (C) levels.

Firmicutes, with a relative abundance ranging from 25.56% to 26.51%, was the dominant phylum in the skin microbiota, followed by Proteobacteria (19.12%–20.71%) and Bacteroidetes (18.29%–19.55%) (Fig. 3A). The dominant order in the skin microbiota was Bacteroidales (15.30%–16.58%), followed by Lachnospirales (6.50%–7.15%) (Fig. 3B). The dominant genus in the skin microbiota was an uncultured bacterium from the Muribaculaceae family (4.93%–5.61%), followed by *Bacteroides* (3.47%–3.84%) (Fig. 3C).

### The spatial variation of skin microbiota in *K. hainanus*

Among the 21 skin samples of *K. hainanus*, the average number of OTUs was highest in the Lingshui population (2356), followed by the Shanglin population (975), and lowest in the Jinxiu population (929). Rarefaction curves approached saturation, indicating that sequencing depth was sufficient for the analyses performed (Fig. S2).

**Figure 4:**
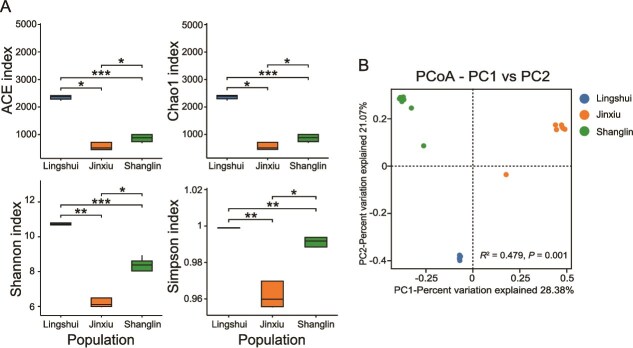
Skin microbiota diversity of *Kurixalus hainanus* among the Lingshui, Jinxiu, and Shanglin populations. (A) Alpha diversity based on ACE, Chao 1, Shannon, and Simpson indices. Differences were tested by Kruskal–Wallis test followed by pairwise Wilcoxon rank-sum tests with Benjamini–Hochberg FDR correction. (B) PCoA based on bray-Curtis distances. PERMANOVA was used to assess differences among populations. Asterisks indicate significant differences. ^*^P < .05, ^**^P < .01, ^***^P < .001.

We observed significant differences in the alpha diversity of skin bacteria among the three populations (Kruskal–Wallis test: *P* < .05, Fig. 4A). The ACE, Chao1, Shannon, and Simpson indices of the skin microbiota in the Lingshui population were significantly higher than those in the other two populations, followed by the Shanglin population and then the Jinxiu population (Wilcoxon rank-sum tests: all *P* < .05, Fig. 4A). For beta diversity of the skin microbiota, PCoA analysis illustrated the variation in skin microbial communities along the first two principal coordinates (PC1 and PC2), which accounted for 28.38% and 21.07% of the total variation, respectively (Fig. 4B). Samples from the three populations formed distinct clusters with no overlap, indicating significant differences in skin microbial community composition among the three locations. The PERMANOVA analysis further confirmed that the geographical location explained 47.9% of the total variation in skin microbial communities, and this effect was statistically significant (bray-curtis: *R*^2^ = 0.479, *P* = .001, Fig. 4B).

**Figure 5:**
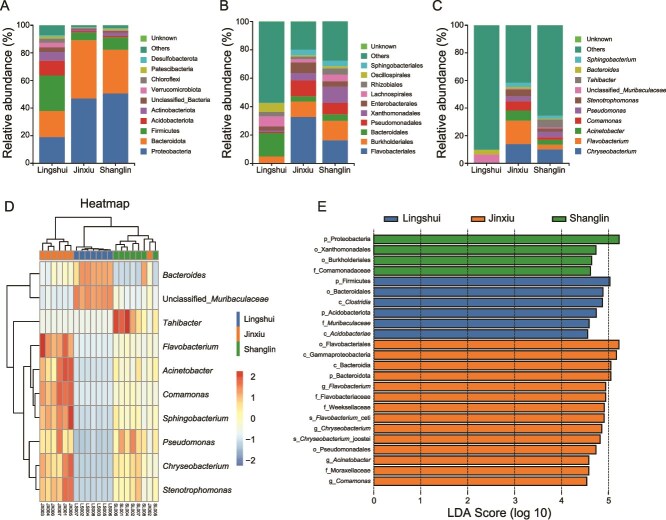
Relative abundance of skin microbiota of *Kurixalus hainanus* among the Lingshui, Jinxiu, and Shanglin populations at the phylum (A), orders (B), and genus (C) levels. (D) Genus-level abundance clustering heatmap. (E) LEfSe analysis of skin microbiota composition (LDA score > 4.5, *P* < .05).

At the phylum level, the Lingshui population was dominated by Firmicutes (25.68%), Proteobacteria (19.01%), Bacteroidota (18.90%), Acidobacteriota (10.76%), and Actinobacteriota (6.33%) (Fig. 5A). The dominant phyla in the Jinxiu population (JX) and the Shanglin population (SL) were also Proteobacteria (JX: 47.01%, SL: 50.63%), Bacteroidota (JX: 42.13%, SL: 31.56%), and Firmicutes (JX: 5.72%, SL: 9.21%) (Fig. 5A). However, there were differences in the specific proportions of each phylum between the Jinxiu and Shanglin populations, reflecting the regional characteristics of microbial composition at the large-scale taxonomic unit level. At the order level, the Lingshui population was dominated by Bacteroidales (16.54%), Lachnospirales (7.10%), and Oscillospirales (6.24%) (Fig. 5B). The dominant order in the Jinxiu and Shanglin populations was Flavobacteriales (JX: 32.74%, SL: 16.43%), Burkholderiales (JX: 10.82%, SL: 13.56%), and Pseudomonadales (JX: 11.14%, SL: 8.00%) (Fig. 5B). Focusing on the genus level, the regional specificity was further highlighted. In the Lingshui population, the proportion of the "Others" group was extremely high, and the dominant genera were relatively vague (Fig. 5C). The Jinxiu population was enriched with genera such as *Chryseobacterium* (13.94%) and *Flavobacterium* (16.96%) (Fig. 5C). The Shanglin population had the specific distribution of genera such as *Tahibacter* (5.68%) (Fig. 5C). Combined with genus-level abundance clustering analysis of the heatmap and clustering tree, the microbial genera from different locations exhibited distinct abundance clustering patterns (Fig. 5D). For example, *Tahibacter* and *Pseudomonas* showed high abundance in samples from the Shanglin population (Fig. 5D). In contrast, genera such as *Bacteroides* and unclassified Muribaculaceae displayed relatively high abundance aggregation in the Lingshui population, whereas several other genera (eg, *Chryseobacterium* and *Comamonas*) were presented at low abundance (Fig. 5D). The Jinxiu population was characterized by a distinct assemblage, with genera such as *Flavobacterium* and *Stenotrophomonas* showing relatively elevated abundance (Fig. 5D). This genus-level abundance clustering pattern clearly and quantitatively reflects regional differentiation in the skin microbiota across locations, as evidenced by the correlated abundance of specific microbial groups. From the perspective of column clustering, samples from the Lingshui, Jinxiu, and Shanglin populations were clustered into separate, well-defined branches, revealing a high degree of within-population consistency in genus-level abundance patterns (Fig. 5D).

LEfSe analysis (|LDA| > 4.5) across the three populations showed that a total of 6 biomarkers were identified in the Lingshui population, 14 in the Jinxiu population, and 4 in the Shanglin population (Fig. 5E). Firmicutes, Clostridia, and Bacteroidales were enriched in the Lingshui population. Flavobacteriales and Gammaproteobacteria were enriched in the Jinxiu population, and Proteobacteria was enriched in the Shanglin population (Fig. 5E).

### Component identification of volatile secretions from *K. hainanus*

A total of 14 chemical compounds were detected in the Lingshui population, of which 13 were identified as volatile organic compounds (VOCs) (Table 1). These VOCs were classified into five categories based on chemical structure, including seven fatty alcohols, three carbonyl compounds, one alkane compound, one benzothiazole compound, and one benzene derivative. A total of 16 chemical compounds were detected in the Jinxiu population, 14 of which were identified as VOCs. These VOCs were primarily composed of six fatty alcohols, four carbonyl compounds, three terpenoids, and one benzothiazole compound (Table 1). The Shanglin population contained 11 chemical compounds, among which 9 VOCs were identified, mainly consisting of 4 fatty alcohols, 3 carbonyl compounds, 1 alkane compound, and 1 benzothiazole compound (Table 1). Among the three populations, the Jinxiu population shared the most compound types with the other two populations. The compounds shared among the three populations were 1,2-benzisothiazole, 1-nonanal, 1-nonanol, 2-ethylhexan-1-ol, decanal, octan-1-ol, and octanal (Table 1). 1-hexanol, 1-octen-3-ol, 2,6,10-trimethyldodecane, and diisobutyl phthalate were exclusively detected in the Lingshui population, while L-menthol was only present in the Jinxiu and Shanglin populations (Table 1). Interestingly, three terpenoids were uniquely found in the Shanglin population: D-limonene, 2-(4-methyl-3-cyclohexenyl)-2-propanol, and β-caryophyllene (Table 1).

**Table 1:** Volatile secretions identified from the skin of *Kurixalus hainanus* in the Lingshui, Jinxiu, and Shanglin populations.

Compounds	Class/subclass	Populations		
		Lingshui	Jinxiu	Shanglin
(4R)-1-methyl-4-(1-methylethenyl)cyclohexene	Monoterpenoids		√	
1,1-Diethoxyethane	Carbonyl compounds		√	
Diisobutyl phthalate	Enzene and substituted derivatives	√		
1,2-benzisothiazole	Benzothiazoles	√	√	√
1-Decanol	Fatty alcohols	√		
1-Hexanol	Fatty alcohols	√		
1-Heptanol	Fatty alcohols		√	
1-Nonanal	Carbonyl compounds	√	√	√
1-nonanol	Fatty alcohols	√	√	√
1-Octen-3-ol	Fatty alcohols	√		
1-p-menthen-8-ol	Monoterpenoids		√	
2,6,10-Trimethyldodecane	Alkanes	√		
2-Ehylhexan-1-ol	Fatty alcohols	√	√	√
(3Z)-3-nonen-1-ol	Fatty alcohols	√	√	
Decanal	Carbonyl compounds	√	√	√
Decane	Alkanes			√
β-Caryophyllene	Sesquiterpenoids		√	
L-Menthol	Fatty alcohols		√	√
1-Octanol	Fatty alcohols	√	√	√
Octanal	Carbonyl compounds	√	√	√
Unknown1	-	√		
Unknown2	-			√
Unknown3	-		√	√
Unknown4	-		√	

√ indicates presence of the compound.

## Discussion

In the present study, we investigated the temporal and spatial variation in skin microbiota of *K. hainanus* and characterized population-level differences in skin volatile secretion composition. We found that ACE and Chao1 richness indices differed significantly among the four months, whereas Shannon and Simpson diversity indices remained stable, likely reflecting the greater sensitivity of ACE and Chao1 to rare taxa [50]. PCoA showed no significant temporal changes in skin microbiota composition across the four months, with extensive overlap among samples from different months, indicating a relatively stable community structure. We also compared the relative abundance of core and rare taxa and found that core taxa maintained relatively stable total abundance across time, whereas rare taxa exhibited significant temporal fluctuations. In addition, all genera showing significant differences among months exhibited relative abundances below 1.2%. Taken together, these results suggest that the skin microbiota remained largely stable over time, with consistent contributions from core taxa, while temporal variation was primarily driven by rare taxa. A similar pattern was found in a study of Sierra Nevada yellow-legged frog (*Rana sierrae*), which showed that low-abundance amplicon sequence variants (ASVs) exhibited the highest turnover rates over time compared to intermediate or high abundance ASVs [51].

Such temporal pattern may reflect the combined effects of stable host-microbe symbiosis and subtle environmental variation. The dominant microbiota forms a stable symbiotic core that maintains essential community functions, and the stability of skin microbiota has been shown to be critical for host health [19]. Disruption of this balance can weaken pathogen resistance, and even normally benign skin bacteria may become opportunistic pathogens [19, 52]. For example, Jiang et al. [53] reported that skin microbiota composition is closely associated with skin ulceration in crocodile lizards (*Shinisaurus crocodilurus*), with both bacterial and fungal communities showing pronounced shifts on ulcerated skin. In contrast, seasonal environmental changes in Hainan Province—characterized by a gradual increase in temperature and precipitation from April to July–May preferentially influence rare microbial taxa, which are generally considered more sensitive to subtle external fluctuations and microhabitat variation than dominant microbiotas, thereby contributing to the observed temporal turnover of these low-abundance taxa. In addition, the composition and quantity of frog skin secretions can vary over time, potentially altering the skin microenvironment and acting as selective filters for environmental bacteria [54, 55]. For instance, the antimicrobial capacity of skin secretions in the diskless-fingered odorous frog (*Odorrana grahami*) exhibits clear temporal variation [30]. Mechanistically, skin secretions may damage some types of bacterial cell membranes [56], whereas some microbes may be selectively attracted to host-derived compounds [57]. Although these mechanisms cannot be disentangled directly in this study, the observed turnover of rare taxa is likely driven by their heightened sensitivity to both environmental fluctuations and host-mediated chemical changes.

Among taxa with relatively stable abundance, the dominant phyla were Firmicutes, Proteobacteria, and Bacteroidetes. These phyla have been widely documented as dominant in several anuran species, including the Northeast China Brown Frog (*Rana dybowskii*) [58], the Tibetan toad (*Bufo tibetanus*), the Plateau Brown Frog (*Rana kukunoris*), the Xizang AIpine Toad (*Scutiger boulengeri*), and the Tibetan frog (*Nanorana pleskei*) [59], suggesting a conserved core skin microbiota across amphibians. *Lactobacillus* was identified as one of the dominant genera. As a core dominant genus in the vagina of human females, it has been confirmed to exert mucosal protective effects in mammalian mucosal systems [60, 61]. It raises the possibility that *Lactobacillus* may play potential protective or pH-regulating roles in amphibians, though functional validation is required.

In contrast to the relatively subtle temporal variation, pronounced geographical differentiation in skin microbiota composition was observed among the Lingshui, Jinxiu, and Shanglin populations. The PCoA revealed clear separation of the three populations in terms of skin microbiota composition and abundance. The PERMANOVA analysis supported that these differences were statistically significant, and geographical factors had high explanatory power for this differentiation. This finding is consistent with the results of previous studies on the Coqui frog (*Eleutherodactylus coqui*) [62, 63] and other amphibians [64, 65]. Individuals from the Lingshui population exhibited significantly higher microbial richness and diversity compared to those from the Jinxiu and Shanglin populations. This may be associated with the tropical monsoon climate of Lingshui, which is characterized by high temperature, high humidity, and abundant precipitation [66, 67]. This stable, warm, and humid environment may provide favorable growth conditions for thermophilic and hygrophilic microorganisms [68]. These environmental variables may contribute to shaping the microbiota, but their causal effects remain to be experimentally verified. A growing body of evidence indicates that habitat can strongly influence microbiome structure and composition [51, 69, 70], while such alteration of the microbiome may constitute a fast-response mechanism of the host to environmental changes [71]. Ziegler et al. [72] defined microbiome flexibility as the potential for dynamic restructuring of a host’s microbial community in response to environmental changes. This flexibility is presumed to enable the metaorganism to adapt rapidly to environmental changes, but it may also lead to the loss of functionally important microbial associates [72]. Thus, we should pay more attention to the skin microbiota of amphibians during reintroduction programs. Exposure to the microbiota of the target site can be introduced gradually to facilitate adaptation, while sudden environmental changes should be avoided to minimize the loss of core microbiota.

Regarding the composition of skin microbiota, the three populations differed in the relative proportions of major phyla, and these geographical differences became even more pronounced at the genus level, highlighting strong regional specificity. Such genus-level differentiation is likely driven by habitat heterogeneity among populations. *Tahibacter* and *Pseudomonas* were significantly enriched in the Shanglin population. A study investigating the correlation between soil microorganisms and soil metabolites demonstrated that *Tahibacter* and *Pseudomonas* exhibit a positive correlation with soil lipids [73]. Therefore, we speculate that the enrichment of these two genera in Shanglin may be associated with the habitat’s characteristics of abundant organic matter and interweaved aquatic-terrestrial environments. *Flavobacterium* and *Stenotrophomonas* were relatively aggregated in the Jinxiu population, which may match the cool mountain environment, as a study that isolated and identified them while isolating psychrotolerant bacteria from the wild flora of the Andes Mountains and Chilean Patagonia [74]. By contrast, in the tropical habitat of Lingshui, *Bacteroides* and unclassified Muribaculaceae showed relatively higher abundance, whereas genera such as *Chryseobacterium* showed low abundance. This distribution pattern may reflect selective adaptation of microbiota to persistently high temperature and humidity [75]. When interpreting these geographical patterns, it is also important to consider differences in sampling procedures among populations. Skin microbiota from the Lingshui population were collected directly in the field, whereas samples from the Jinxiu and Shanglin populations were obtained after frogs were transported to the laboratory in Sichuan Province. To minimize potential disturbance, frogs were transported together with nearby vegetation and sampled immediately upon arrival; nevertheless, minor differences between field and laboratory conditions may have introduced subtle biases in microbial composition.

Overall, skin secretions were largely similar among the three populations, although each population exhibited a small number of unique compounds. The compounds with the highest detection frequencies included nonanal, decanal, 2-ethyl-1-hexanol, 1-nonanol, and 1-octanol, suggesting that these substances constitute the core components of skin volatiles. Among them, decanal and 1-octanol have been demonstrated to function in defense and alarm signaling in insects. For instance, decanal has been identified as a defensive compound in the secretions of the tick (*Haemaphysalis leachi*) [76]. Similarly, 1-octanol is a major component of honeybee alarm pheromones and also exhibits mosquito-repellent activity [77]. These findings raise the possibility that decanal and 1-octanol may serve comparable defensive or signaling functions in frogs. Previous studies with *K. hainanus* showed that disturbance odors induce avoidance behavior in females and reduce males’ call rate [37, 38]. Accordingly, we speculate that decanal and 1-octanol may contribute to the chemical basis of these disturbance odors.

In addition, the core skin volatile compounds, decanal and 1-octanol, were consistently associated with a high abundance of Proteobacteria across all populations. Taxa such as *Pseudomonas* and *Stenotrophomonas* are well-documented for their ability to synthesize or degrade long-chain VOCs [78]. We therefore hypothesize that these core secretions may function as a chemical sieve, selectively promoting the colonization of beneficial Proteobacteria while inhibiting competitors. It is worth noting that three terpenoid compounds—two monoterpenes and one sesquiterpene—were detected exclusively in individuals from the Shanglin population. Previous studies have reported monoterpenes and sesquiterpenes in the volatile secretions of African reed frogs (Hyperoliidae) and the Australian green treefrog (*Litoria caerulea*), and have demonstrated that these compounds originate primarily from environmental sources rather than endogenous synthesis [48, 79]. These findings suggest that environmental inputs may contribute to population-specific variation in frog skin volatile secretions. Meanwhile, microbial metabolites represent a potential source of individual volatile secretions [27]. Therefore, future studies could aim to identify the specific microbial taxa and environmental compounds involved, and to test how these factors influence the chemical signaling functions of frog skin volatiles. Because temporal variation in skin secretions was not assessed in this study, we cannot exclude the possibility that shifts in rare microbial taxa are associated with changes in chemical secretions over time. One limitation is that the low secretion yield from individual frogs required pooling samples for volatile analysis, preventing robust quantitative comparisons among populations. Future studies using improved sampling and detection methods, ideally with synchronized and individual-matched designs, will be needed to clarify microbiota–secretion relationships.

In summary, our study shows that the skin microbiota of *K. hainanus* is characterized by a temporally stable core community, with temporal variation driven primarily by rare taxa, and pronounced geographical differentiation among populations. This pattern may help maintain essential skin-associated functions while allowing flexible responses to short-term environmental variation. Furthermore, habitat-specific conditions appear to shape divergent skin microbial assemblages, which may, in turn, contribute to host adaptation to local environments. Together, these findings highlight a dynamic balance between environmental pressures and the structural stability of skin microbial communities. Understanding this balance is relevant not only for conservation efforts such as species reintroduction, but also for anticipating how ongoing environmental change—such as climate shifts and habitat alteration—may disrupt host–microbiota associations.

## Supplementary Material

ycag125_Supplemental_Files

## Data Availability

The raw sequences were deposited in the NCBI Sequence Read Archive (SRA) database under BioProject accession number PRJNA1399319.
