# Supplementary material for: Temporal stability and geographical divergence of skin microbiota in a treefrog: insights from microbial communities and volatile secretions
Source: ISME Commun. 2026 May 7;6(1):ycag125. doi: 10.1093/ismeco/ycag125 (PMC13219742; doi:10.1093/ismeco/ycag125)
Supplement: ycag125_Supplemental_Files [file ycag125_supplemental_files.zip › Supplemental Material_ycag125.pdf]

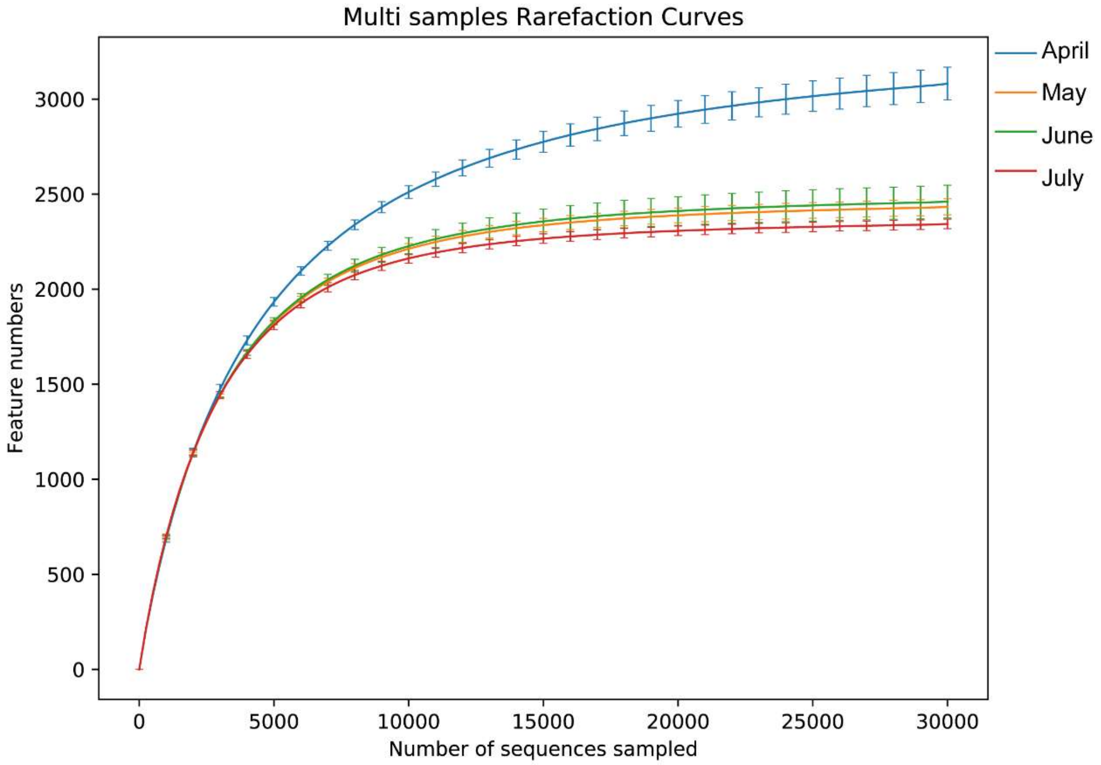

Figure S1 Rarefaction curves of samples collected in different months. Different colors represent samples collected in April, May, June, and July.

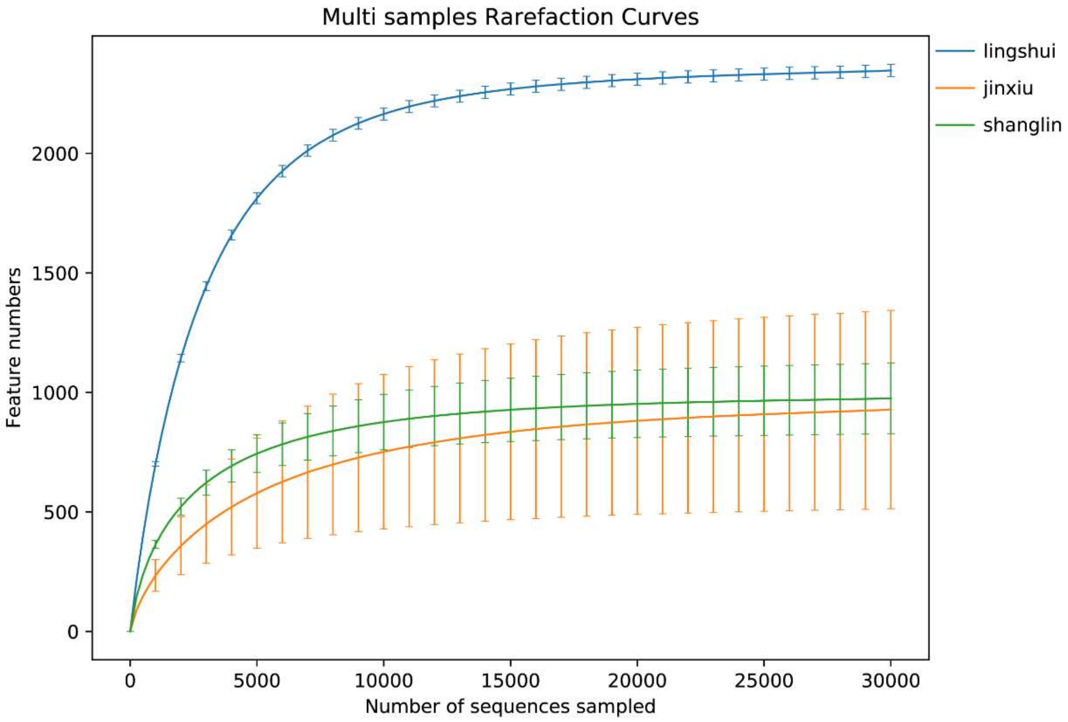

Figure S2 Rarefaction curves of samples collected in different months. Different colors represent samples collected from Lingshui, Jinxiu, and Shanglin.

Table S1 Overview of sample information and sequencing read processing results.
